# Supplementary material for: High-Frequency Irreversible Electroporation (H-FIRE) Induced Blood–Brain Barrier Disruption Is Mediated by Cytoskeletal Remodeling and Changes in Tight Junction Protein Regulation
Source: Biomedicines. 2022 Jun 11;10(6):1384. doi: 10.3390/biomedicines10061384 (PMC9220673; doi:10.3390/biomedicines10061384)
Supplement: Supplementary file 1 [file biomedicines-10-01384-s001.zip › biomedicines-1738405-Supplementary.pdf]

Gene Expression Scatterplot: Sham Control vs. 1h Post-treatment

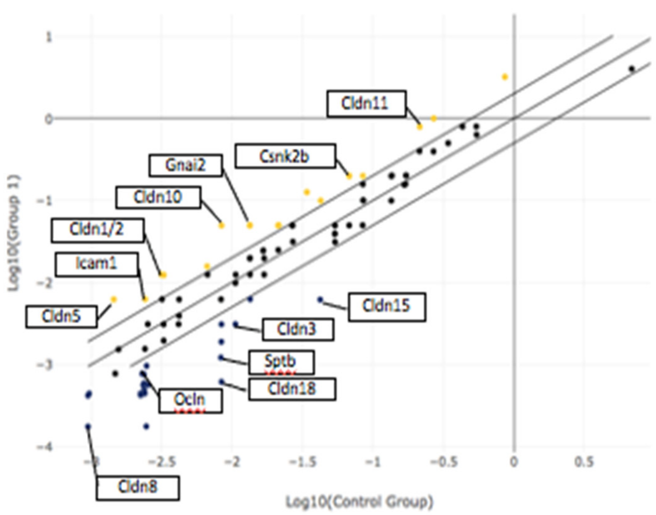

Gene Expression Scatterplot: Sham Control vs. 24h Post-treatment

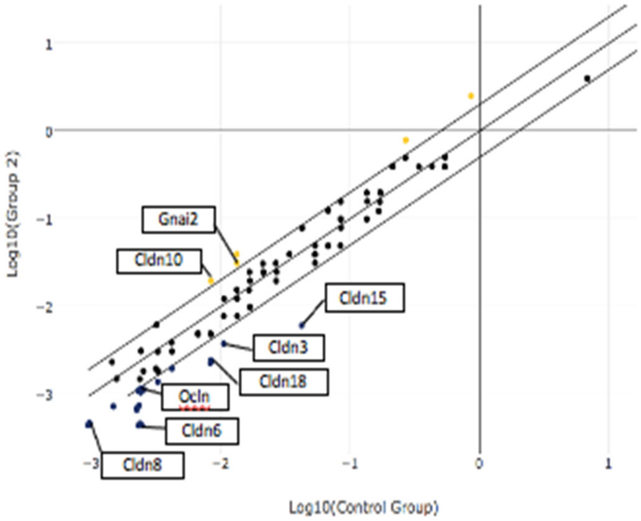

Gene Expression Scatterplot: Sham Control vs. 48h Post-treatment

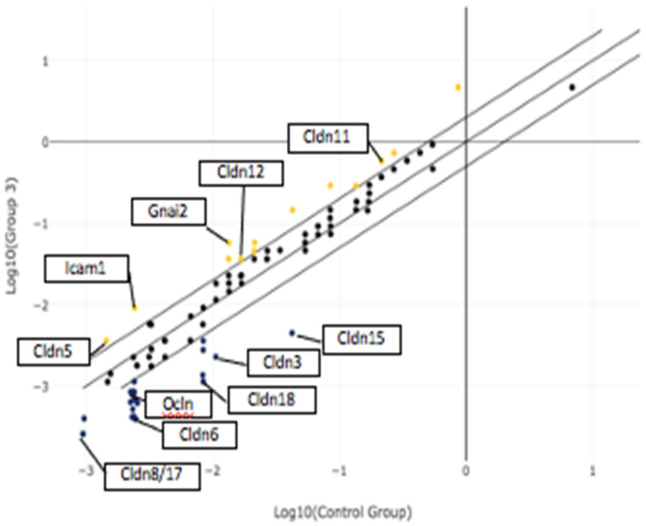

Gene Expression Scatterplot: Sham Control vs. 72h Post-treatment

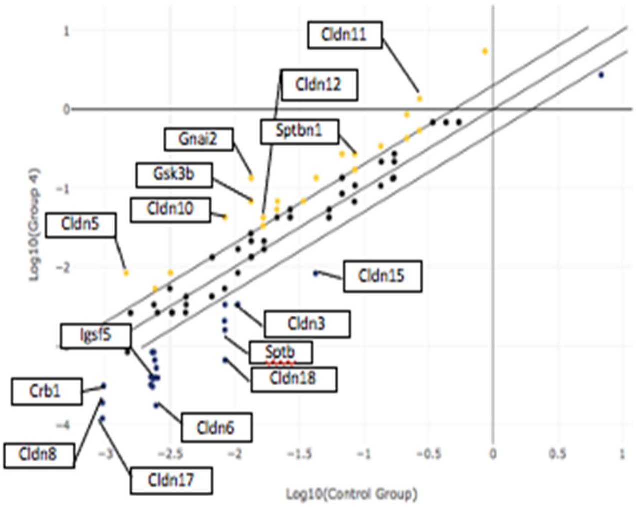

Gene Expression Scatterplot: Sham Control vs. 96h Post-treatment

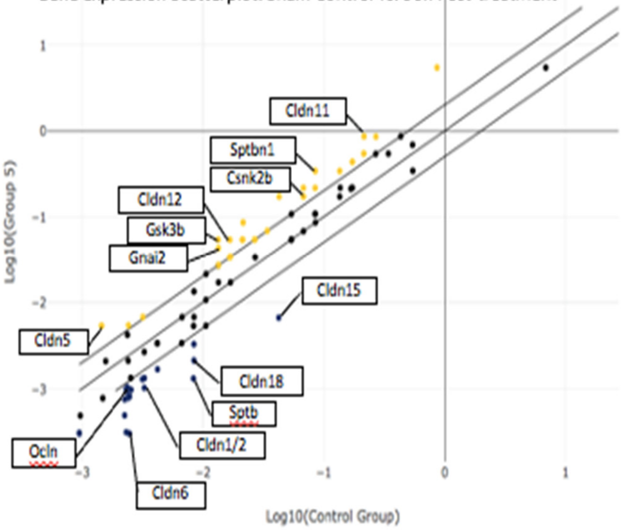

Figure S1: Scatterplots summarizing GeneGlobe results from gene expression and pathway analysis using Tight Junction RT2 Profiler PCR Array over time compared to sham controls. Genes with significant increases in expression relative to sham controls are represented by yellow dots. Genes with significant decreases in expression relative to sham controls are represented by blue dots. Genes without significant dysregulation when comparing treatment groups to sham controls are represented by black dots. A decrease in the quantity of upregulated genes was observed between 1- and 24-hours post-treatment followed by a gradual increase over time.

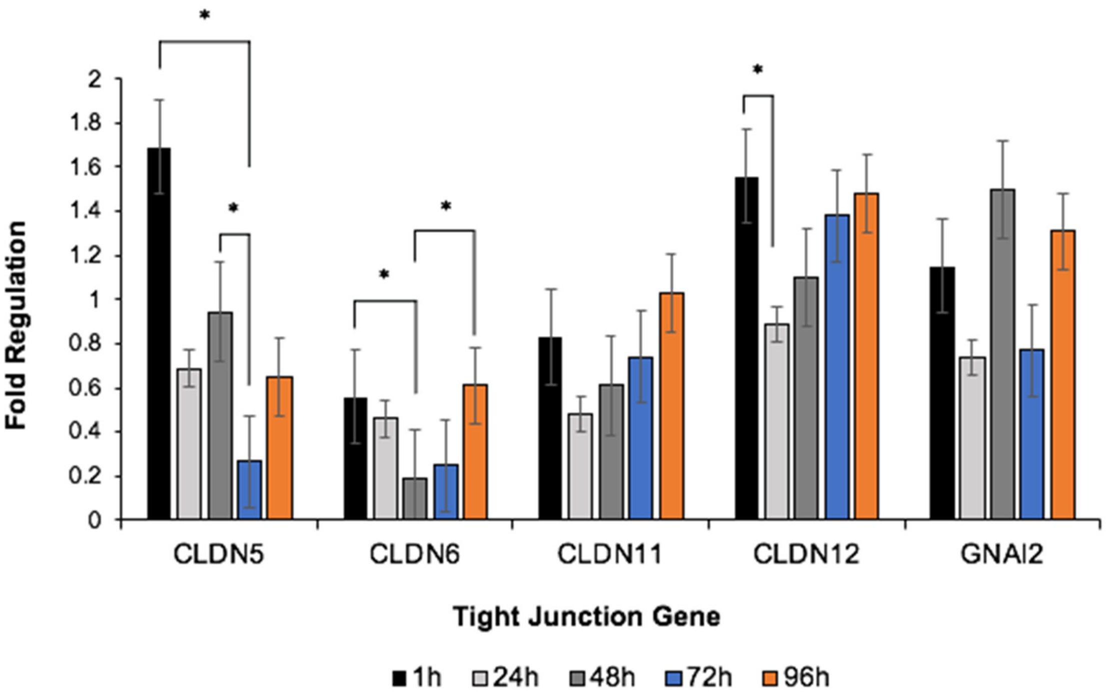

Figure S2: Results from analysis of variance (ANOVA) demonstrating significant differences in gene expression between time points for *CLdn5*, *Cldn6* and *Cldn12*. * $p < 0.05$
